# Understanding the complex role of exosomes in intestinal ischemia-reperfusion injury: from pathogenesis to protection

**DOI:** 10.3389/fphar.2025.1533628

**Published:** 2025-04-15

**Authors:** Qin Ye, Zi-Hang Yu, Liang Nie, Fei-Xiang Wang, Guo Mu, Bin Lu

**Affiliations:** ^1^ Department of Anesthesiology, Zigong Fourth People’s Hospital, Zigong, Sichuan, China; ^2^ Department of Anesthesiology, Fushun County People’s Hospital, Zigong, Sichuan, China; ^3^ Department of Anesthesiology, The Affiliated Hospital, Southwest Medical University, Luzhou, Sichuan, China

**Keywords:** exosomes, ischemia-reperfusion injury, intestinal ischemia-reperfusion injury, intestinal injury, multiorgan injury

## Abstract

Extracellular vesicles, which are predominantly classified into ectosomes and exosomes, are released by all cells under both physiological conditions and in response to acquired pathological states. Exosomes demonstrate multifaceted functions: they regulate cellular homeostasis through the elimination of redundant or detrimental intracellular components, function as mediators in intercellular signaling pathways, and serve as potential vectors for both diagnostic and therapeutic applications. Intestinal ischemia-reperfusion injury (IRI), a prevalent form of tissue and organ injury in surgical settings, has been extensively investigated. Emerging evidence indicates a crucial relationship between exosomes and intestinal IRI, specifically regarding how exosomes derived from either intestinal tissue or distant organs can modulate the pathophysiological progression of intestinal IRI. This review systematically evaluates the mechanistic roles of exosomes in intestinal IRI and their involvement in post-intestinal IRI multiple organ dysfunction, aiming to establish a theoretical foundation for therapeutic interventions and future research directions.

## Graphical Abstract

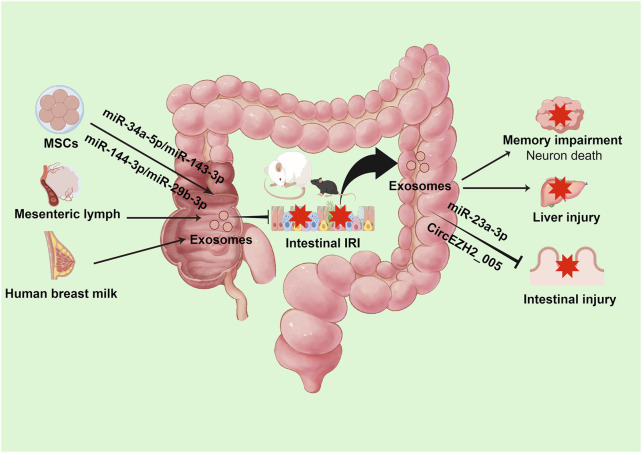

## 1 Introduction

The release of extracellular vesicles (EVs) is a universal cellular process observed in both prokaryotic and eukaryotic cells under physiological conditions and in response to pathological stimuli, representing a fundamental aspect of cellular function. The classification and functional characteristics of EVs merit extensive investigation due to their critical role as mediators of intercellular material exchange. EVs are predominantly classified into two distinct categories: ectosomes and exosomes, each exhibiting unique morphological, dimensional, and functional characteristics. Exosomes, a specialized subclass of EVs characterized by an average diameter of approximately 100 nm, demonstrate distinctive capabilities in intercellular communication (Cocucci and Meldolesi, 2015; Bebelman et al., 2018; Willms et al., 2018). These nanovesicles harbor a diverse cargo of biomolecules, encompassing nucleic acids, proteins, lipids, amino acids, and metabolites, which serve as molecular signatures of their cellular origin and provide crucial insights into cellular functions and disease pathogenesis (Ding et al., 2024; Pathan et al., 2019; van Balkom et al., 2015). Exosomes exhibit dual functionality in cellular physiology: they maintain cellular homeostasis through the selective elimination of redundant or potentially detrimental cellular components while simultaneously serving as sophisticated mediators of intercellular signaling in various physiological and pathological processes. Their distinctive biological properties have positioned exosomes as promising candidates for diagnostic biomarkers and therapeutic delivery vehicles, potentially revolutionizing approaches to precision medicine (Kalluri and LeBleu, 2020).

The maintenance of oxygen homeostasis, fundamentally dependent on adequate tissue perfusion, is essential for organismal viability (Lier et al., 2018). Ischemia-reperfusion injury (IRI), a complex pathophysiological cascade first documented in 1952, represents a significant clinical challenge with substantial implications for patient outcomes (Sunder-Plassmann, 1952). The pathogenesis of IRI initiates with arterial occlusion, resulting in tissue hypoperfusion and subsequent cellular dysfunction due to metabolic substrate deficiency and oxygen deprivation. Paradoxically, the restoration of blood flow often intensifies tissue injury rather than facilitating recovery, ultimately promoting cellular necrosis (Wu et al., 2018; Jiménez-Castro et al., 2019). In surgical contexts, intestinal IRI represents a prevalent form of tissue injury associated with various pathological conditions, including intestinal volvulus, intussusception, strangulated hernia, acute mesenteric ischemia, shock, and trauma. Furthermore, specific surgical interventions, particularly intestinal resection and transplantation procedures, may precipitate intestinal IRI (Liao et al., 2022). Consequently, the development of effective therapeutic strategies targeting post-intestinal IRI microcirculatory dysfunction and subsequent multi-organ injury remains an area of intense scientific investigation.

Extensive experimental evidence has established the fundamental role of exosomes in intestinal IRI pathophysiology. These studies have demonstrated that exosomes, whether derived from intestinal tissue or distant organs, exert significant modulatory effects on both intestinal IRI progression and secondary multiple organ dysfunction (Senda et al., 2023; Zhao et al., 2022; Chen et al., 2021). This emerging understanding of exosome biology in intestinal IRI presents novel opportunities for therapeutic intervention. The present review synthesizes current evidence regarding the mechanistic roles of exosomes in intestinal IRI and systematically examines their contribution to post-injury multiple organ dysfunction. Through comprehensive analysis of exosome-mediated pathophysiological processes in intestinal IRI, we aim to establish a robust theoretical framework to guide therapeutic innovation and future research initiatives.

## 2 Biogenesis pathway of exosomes

**FIGURE 1:**
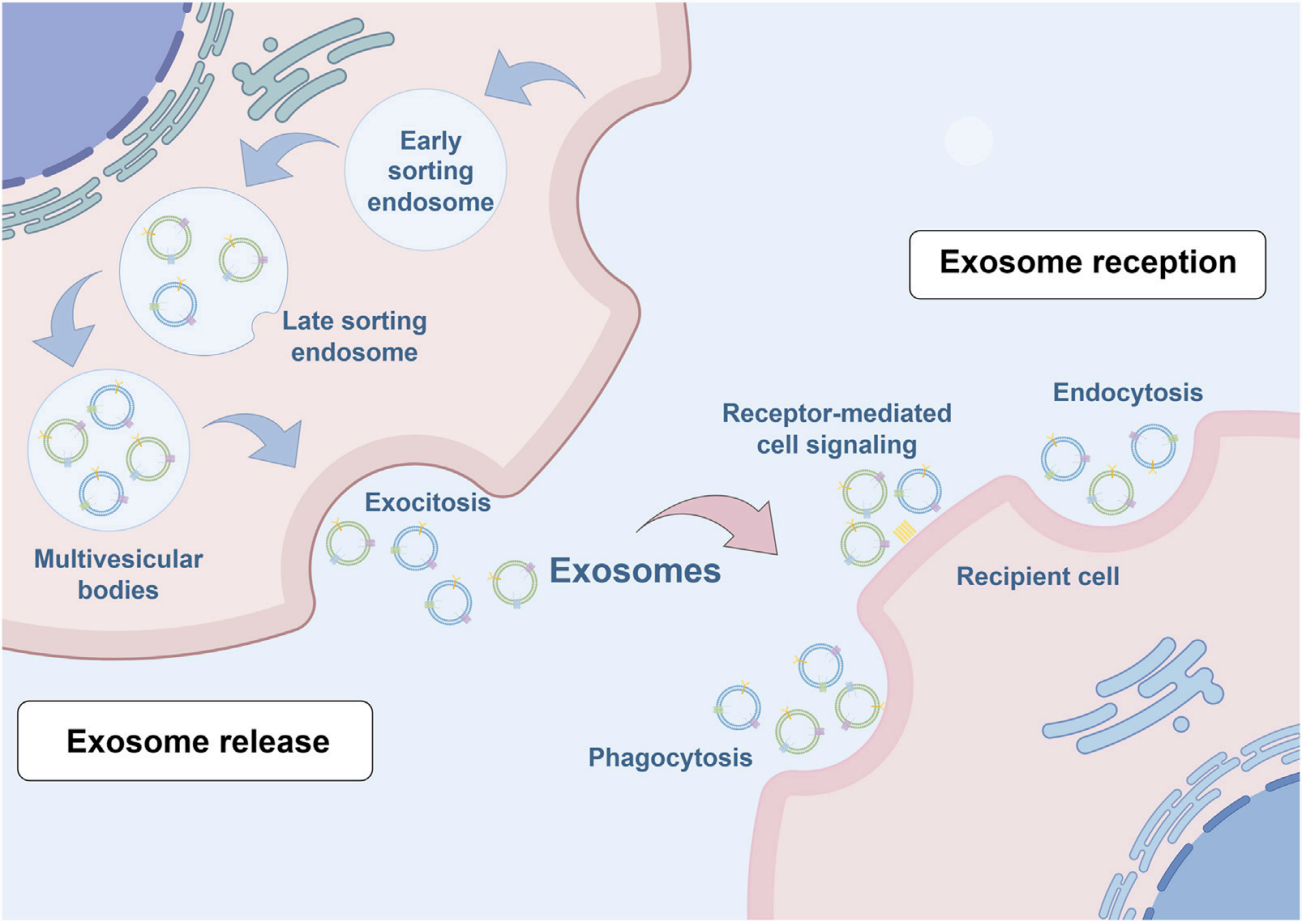
Schematic illustration of exosome biogenesis. Exosome formation encompasses a series of highly orchestrated cellular processes. The initial step involves plasma membrane invagination, forming characteristic cup-shaped structures that incorporate cell surface proteins and their associated soluble components. These structures are essential for early sorting endosome development, a process regulated by both the Golgi apparatus and endoplasmic reticulum. The maturation process continues as early endosomes progress to late sorting endosomes under the precise control of the endosomal sorting complex required for transport machinery and associated transport proteins. Subsequently, secondary invagination occurs, leading to the formation of multivesicular bodies (MVBs). These specialized compartments serve as repositories for diverse bioactive molecules, which are ultimately released into the extracellular space through MVB-plasma membrane fusion events, facilitating intercellular communication and molecular exchange.

The biogenesis of exosomes occurs through an intricately orchestrated process characterized by the invagination of double-layered membranes and subsequent formation of multivesicular bodies (MVBs), particularly those harboring intraluminal vesicles (ILVs). ILVs, serving as exosomal precursors, are ultimately secreted into the extracellular space following MVB-plasma membrane fusion and exocytosis, with characteristic dimensions ranging from 40 to 160 nm. The initial stage involves primary plasma membrane invagination, generating a cup-shaped structure that incorporates both membrane-associated proteins and soluble proteins from the extracellular microenvironment. This critical step facilitates early sorting endosome (ESE) formation, wherein nascent ESEs may undergo fusion with pre-existing ESEs under specific physiological conditions, resulting in enhanced content diversity and functional capacity. The trans-Golgi network and endoplasmic reticulum contribute fundamentally to ESE biogenesis and cargo regulation through the provision of essential membrane constituents and proteins while orchestrating cargo sorting and packaging processes. Subsequently, under precise molecular regulation mediated by the endosomal sorting complex and associated transport proteins, early endosomes undergo maturation into late sorting endosomes (LSEs). Following secondary invagination, LSEs culminate in MVB formation, which serve as crucial cellular compartments for the storage and regulated release of bioactive molecules via membrane fusion events, thereby facilitating intercellular communication and molecular exchange (Kalluri and LeBleu, 2020; Zhang et al., 2020). Figure 1 provides a schematic representation of exosome biogenesis.

Notably, exosome heterogeneity also represents a critical focus of contemporary research, reflecting diverse parameters including dimensional variance, cargo composition, recipient cell responses, and cellular origin. Size heterogeneity may arise from asymmetric invagination of MVB limiting membranes, resulting in differential fluid and solid content distribution and consequent dimensional variations. Additionally, methodological variations in EV isolation procedures may contribute to observed size disparities (Kalluri and LeBleu, 2020). Current classification systems predominantly utilize cellular origin as the primary criterion, though this approach presents inherent limitations in comprehensively characterizing distinct exosome subtypes and their functional applications. Future taxonomic approaches might benefit from multi-parametric analysis incorporating factors such as lipophilicity, which reflects membrane compositional characteristics potentially correlating with biological activity. Moreover, biodistribution patterns and immunogenic properties represent essential classification parameters, providing insights into tissue-specific targeting mechanisms and immune system interactions, respectively (Zhang et al., 2020).

The elucidation of exosome biogenesis reveals a highly coordinated process involving multiple organelle systems and sophisticated molecular regulatory mechanisms. This understanding not only advances our knowledge of intracellular trafficking and membrane dynamics but also presents novel opportunities for therapeutic intervention and diagnostic applications.

## 3 Exosome-mediated regulation of IRI disease progression

Exosomes demonstrate pivotal regulatory functions across diverse IRI-related pathologies, encompassing myocardial, pulmonary, and hepatic manifestations. Mechanistic investigations have elucidated complex signaling cascades and multifaceted molecular mechanisms through which exosomes modulate IRI pathophysiology. Specifically, exosome-associated miRNAs demonstrate crucial regulatory functions in post-myocardial IRI cardiac repair and remodeling processes (Liu et al., 2024). Moreover, exosomes exhibit the capacity to suppress interleukin-18 translation in pulmonary microvascular endothelial cells, thereby preventing ubiquitin-mediated degradation of forkhead box P3 protein in CD4 T cells, promoting regulatory T cell differentiation, and attenuating pulmonary IRI pathogenesis (Gao et al., 2024). Furthermore, exosomes serve as valuable diagnostic biomarkers for hepatic IRI, facilitating early detection and therapeutic intervention (You et al., 2024). These findings substantiate the central regulatory role of exosomes in IRI pathophysiology while highlighting their potential utility in both diagnostic and therapeutic applications.

The intercellular communication capabilities of exosomes facilitate complex interactions between distinct IRI pathologies, establishing mechanistic links between organ-specific manifestations. High-throughput sequencing analyses have identified 63 distinct miRNAs associated with brain, cardiac, renal, and hepatic IRI pathologies. This molecular signature comprises 28 miRNAs associated with ischemic stroke, 30 with myocardial infarction, 19 with renal IRI, and 3 with hepatic IRI. Notably, significant molecular overlap exists between different IRI manifestations, with nine common miRNAs identified between ischemic stroke and myocardial infarction, and eight between renal IRI and myocardial infarction. Two specific miRNAs, miR-146a-5p and miR-124, demonstrate associations with three distinct IRI manifestations, although no single miRNA exhibits universal involvement across all four pathologies (Xin et al., 2022).

These findings underscore the extensive therapeutic potential of exosomes in biomedical applications. Exosomes demonstrate remarkable capacity for attenuating IRI-induced cellular and tissue injury through multiple mechanisms, including immunomodulation, promotion of angiogenesis, and enhancement of tissue regeneration (Liao et al., 2022). Their unique biological properties facilitate both diagnostic applications and targeted therapeutic interventions. The identification of common molecular mediators across different IRI manifestations establishes a robust theoretical framework for therapeutic development (Xin et al., 2022). Significantly, exosome-based targeted therapeutic strategies present unprecedented opportunities for IRI treatment, potentially enabling more precise and efficacious therapeutic approaches while defining new trajectories in biomedical research (Ding et al., 2024). These advances warrant continued investigation into the therapeutic applications of exosomes in IRI pathologies.

## 4 The role of exosomes in intestinal barrier dysfunction and restoration

The intestinal barrier system represents a fundamental structural component that provides critical protection against injury to the intestine and adjacent tissues and organs. The intestinal mucosa and epithelium constitute the primary defense line, establishing a selective barrier between luminal contents and underlying tissues. Additionally, the mucus layer, comprised of mucins secreted by goblet cells, provides further separation between microorganisms and particulate matter from the epithelial surface. Epithelial tissue mediates the selective transport of water and nutrients via complex intercellular junctional assemblies, including tight junctions, adherens junctions, and desmosomes. These structural proteins collectively form the apical junctional complex, which regulates both intercellular communication and paracellular permeability. The tight junction protein network, located at the epithelial cell apex, consists of several protein families, including occludin, claudin, zonula occludens, and adherens junction molecules. These proteins are instrumental in maintaining epithelial barrier integrity, with their expression and distribution patterns notably altered during pathological states characterized by enhanced permeability. Under severe pathological conditions, epithelial injury and cellular death can lead to complete tight junction disruption, facilitating the translocation of macromolecules and viable bacteria. Such circumstances of increased transcellular pathway permeability are accompanied by enhanced paracellular transport. These pathological alterations, encompassing reduced mucus layer thickness, inflammatory amplification, and diminished expression and functionality of epithelial and endothelial tight junction proteins, facilitate bacterial and antigenic translocation into the circulation, subsequently inducing systemic inflammatory responses (Lewis and Taylor, 2020).

Extensive investigations have substantiated the regulatory functions of exosomes in intestinal barrier homeostasis across diverse pathological models, particularly in inflammatory bowel disease and experimental colitis. These studies have elucidated not only the fundamental role of exosomes in barrier function regulation but also their underlying molecular mechanisms (Zhang et al., 2019; Kim et al., 2023; Gao et al., 2022). Notable findings demonstrate that curcumin-derived nanovesicles exhibit potent anti-inflammatory properties in intestinal inflammation. These nanovesicles demonstrate preferential accumulation in inflamed colonic regions and exert significant anti-inflammatory effects in both *in vitro* and *in vivo* experimental systems. Furthermore, they effectively modulate gut microbiota composition and macrophage polarization states, thereby facilitating the restoration of compromised intestinal barrier function. Specifically, these nanovesicles enhance the recovery of barrier integrity through upregulation of tight junction proteins and other barrier-associated markers (Gao et al., 2022). Moreover, in the context of intestinal infections, exosomes also demonstrate significant pathophysiological relevance. For instance, following *fusobacterium* nucleatum infection, infected intestinal epithelial cells secrete exosomes containing miR-129-2-3p, which, upon transfer to uninfected epithelial cells, exacerbate barrier dysfunction and promote experimental colitis progression. Mechanistic studies have revealed that this process primarily involves DNA damage induction through the TIMELESS axis, subsequent activation of the ATM/ATR/p53 signaling cascade, ultimately leading to cellular senescence, barrier dysfunction, and colitis development (Wei et al., 2023).

These findings present intriguing paradoxes that warrant comprehensive investigation. The studies highlight the dual nature of exosomes in intestinal pathophysiology. On one positive aspect, exosomes demonstrate remarkable potential as nanoscale therapeutic carriers, exhibiting efficient accumulation at sites of intestinal injury and effective modulation of gut microbiota dysbiosis. Through mechanisms including tight junction protein restoration, exosomes can significantly ameliorate barrier dysfunction, thereby offering therapeutic benefits in various intestinal disorders. Conversely, exosomes derived from injured intestinal epithelial cells may exert deleterious effects, potentially aggravating barrier dysfunction through DNA damage induction and inflammatory pathway activation, thereby contributing to inflammatory bowel disease pathogenesis. These seemingly contradictory findings underscore the complexity of exosome biology. The observed divergent effects suggest that exosomes from distinct cellular or tissue origins may differentially regulate intestinal barrier function, either promoting intestinal homeostasis or exacerbating tissue injury. Therefore, future investigations into exosome-mediated regulation of intestinal barrier function necessitate careful consideration of cell-type specificity to enhance our understanding of disease pathogenesis and intercellular communication networks. Moreover, these findings also provide novel research directions for identifying bioactive components of therapeutic agents and expanding their clinical applications.

## 5 Dual functions of intestinal-derived exosomes in local IRI and secondary remote organ injury

Accumulating evidence indicates that intestinal-derived exosomes play pivotal roles in disease progression, with their characteristics varying according to distinct intestinal regions. This observation provides novel insights into the mechanistic roles of intestinal exosomes in pathological processes. Notably, murine model studies have demonstrated significant proteomic differences between ileal and colonic epithelial cell-derived exosomes, both in protein composition and functionality. Specifically, ileal epithelial cell-derived exosomes are predominantly enriched in proteins associated with genetic information processing pathways, particularly those mediating bile acid transport, protein synthesis, and post-translational modifications. Conversely, colonic epithelial cell-derived exosomes primarily contain proteins involved in metabolic pathways regulating carbohydrates, fatty acids, amino acids, xenobiotics, and bone metabolism, which are essential for metabolic homeostasis. Further analyses have revealed that these differentially expressed proteins are not only crucial for digestive system functions but also intricately linked to the pathogenesis of various disorders, including infectious diseases, endocrine disorders, and osteoarthritis (Ding et al., 2022). Moreover, subsequent investigations have demonstrated that following trauma/hemorrhagic shock, intestinal epithelial cells release immunomodulatory exosomes into the mesenteric lymphatic system. These exosomes interact with dendritic cells, key orchestrators of adaptive immunity, leading to dendritic cell exhaustion and subsequent immunosuppression (Kojima et al., 2018). This mechanism elucidates the crucial immunoregulatory role of intestinal exosomes following trauma/hemorrhagic shock and its potential implications for disease outcomes.

In the context of intestinal IRI, intestinal tissue exhibits complex physiological responses, including the secretion of injury signal-bearing exosomes into the circulatory system. These exosomes function not only as signaling molecules affecting intestinal repair and regeneration but also as mediators of systemic organ dysfunction. Omics analysis of human intestinal Caco-2 cells has demonstrated that hypoxia-reoxygenation significantly upregulates lipid metabolism-related proteins, coupled with subtle alterations in miRNA profiles and elevated concentrations of unsaturated fatty acids in lysophosphatidylcholine. These findings elucidate the potential impact of IRI-associated exosomal component modifications, particularly in inflammatory cascades, including NF-κB pathway activation (Senda et al., 2023). Furthermore, emerging evidence highlights the role of intestinal exosomes in remote organ injury following intestinal IRI. These exosomes can traverse the circulation and induce M1 macrophage polarization in hepatic tissue, thereby exacerbating post-intestinal IRI hepatic injury (Zhao et al., 2022).

Similarly, in the context of brain injury induced by intestinal IRI, intestinal exosomes demonstrate significant pathophysiological importance. Experimental evidence indicates that intracerebroventricular administration of exosomes from intestinal IRI mice significantly activates microglial cells, resulting in neuronal loss, synaptic instability, and cognitive impairment (Chen et al., 2021). Additionally, intestinal epithelial cells may induce cortical neuronal death following intestinal IRI through the release of paracrine mediators, including exosomal miRNAs involved in apoptosis, necroptosis, and pyroptosis (Hsu et al., 2020). These findings underscore the intestine’s role in mediating post-IRI multi-organ dysfunction through exosome-dependent mechanisms and provide novel perspectives on intestinal-brain interactions. Consequently, therapeutic strategies targeting intestinal exosome secretion or microglial activation may represent promising approaches for neuroprotection following intestinal IRI.

**FIGURE 2:**
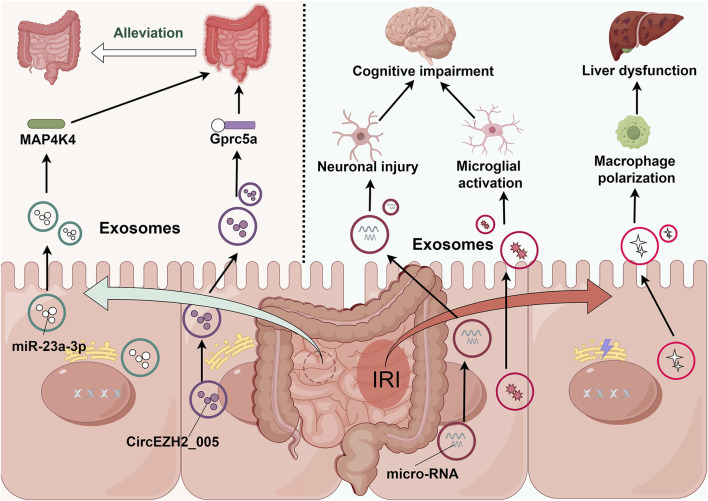
Intestine-derived exosome-mediated signaling cascades in remote organ injury following intestinal IRI. During intestinal IRI, damage-associated exosomes, predominantly released by intestinal epithelial cells, enter systemic circulation and subsequently accumulate in distant organs. These exosomes trigger multi-organ dysfunction through distinct molecular pathways: in the liver, they activate resident macrophages; in the brain, they induce microglial activation and neuronal injury. Paradoxically, these same exosomes demonstrate protective effects through MAP4K4 and Gprc5a signaling pathways, potentially serving as endogenous modulators of intestinal IRI severity.

Notably, post-intestinal IRI exosomes exhibit remarkable biological duality. While potentially exacerbating remote organ injury, they demonstrate protective effects against intestinal IRI. *In vitro* studies have conclusively demonstrated that intestinal epithelial cell-derived exosomes following intestinal IR effectively attenuate injury in hypoxia-high glucose-treated intestinal epithelial cells and mitigate post-IRI intestinal damage. Mechanistic investigations revealed that this protective effect potentially involves miR-23-3p-mediated regulation of MAP4K4 (Yang et al., 2022). Furthermore, recent studies have identified exosomal circEZH2_005 as both a sensitive biomarker for intestinal IRI and a therapeutic mediator operating through Gprc5a regulation (Zhang et al., 2023). (Table 1; Figure 2)

**TABLE 1:** Impact of intestine-derived exosomes on intestinal IRI and secondary remote organ dysfunction.

Exosome origin	Source model	Key findings	References
Intestinal epithelial cells	Human intestinal Caco-2 cells	Characterization of exosomal molecular signatures following intestinal IRI	Senda et al. (2023)
Intestinal epithelial cells	IEC-6 cell line	Exosomal microRNA-mediated cortical neuronal death following intestinal epithelial injury	Hsu et al. (2020)
Intestinal epithelial cells	Rat	Exosome-encapsulated miR-23a-3p attenuates intestinal injury via MAP4K4 targeting	Yang et al. (2022)
Intestinal tissue	Mouse	Exosome-mediated hepatic macrophage polarization contributing to liver dysfunction	Zhao et al. (2022)
Intestinal tissue	Mouse	Exosome-induced microglial activation resulting in cognitive impairment	Chen et al. (2021)
Intestinal tissue	Mouse	CircEZH2_005-regulated Gprc5a signaling pathway mediating intestinal protection	Zhang et al. (2023)

Abbreviations: Gprc5a, G protein-coupled receptor class C group 5 member A; MAP4K4, mitogen-activated protein kinase kinase kinase kinase 4.

These findings not only demonstrate the complex roles of intestinal-derived exosomes in local IRI and secondary remote organ injury but also highlight their functional diversity. This understanding presents several critical research directions, including the temporal sequence of exosome release following intestinal IRI, the initial types of exosomes released, their protective versus deleterious effects, and the mechanisms underlying their differential impact on local versus remote tissue injury. Understanding these aspects may provide novel therapeutic strategies for intestinal diseases.

## 6 Regulatory effects of extra-intestinal exosomes on intestinal IRI

The intestine functions both as an exosome source affecting distant organs and as a recipient of exosomes from various extra-intestinal tissues and organs, enabling precise regulation of intestinal function. Of particular significance is the potential role of cross-species exosomes in intestinal disease pathophysiology. Studies have demonstrated that exosomes isolated from both raw and pasteurized human milk significantly attenuate hypoxia and lipopolysaccharide-induced inflammatory tissue damage compared to controls. *In vivo* investigations have confirmed their efficacy in ameliorating mucosal injury, reducing inflammatory responses, and modulating mucus secretion in necrotizing enterocolitis (Miyake et al., 2020). Additionally, human umbilical cord mesenchymal stem cell (MSC)-derived exosomes containing specific microRNAs, notably miR-378a-5p, demonstrate significant potential in suppressing NLRP3 inflammasome activation, thereby reducing pyroptosis and preventing colitis development (Cai et al., 2021). Furthermore, plant-derived nanoparticles with exosome-like properties, when integrated through intestinal microbiota-dependent mechanisms, modulate gut microbiota composition and enhance intestinal barrier integrity (Teng et al., 2018).

**FIGURE 3:**
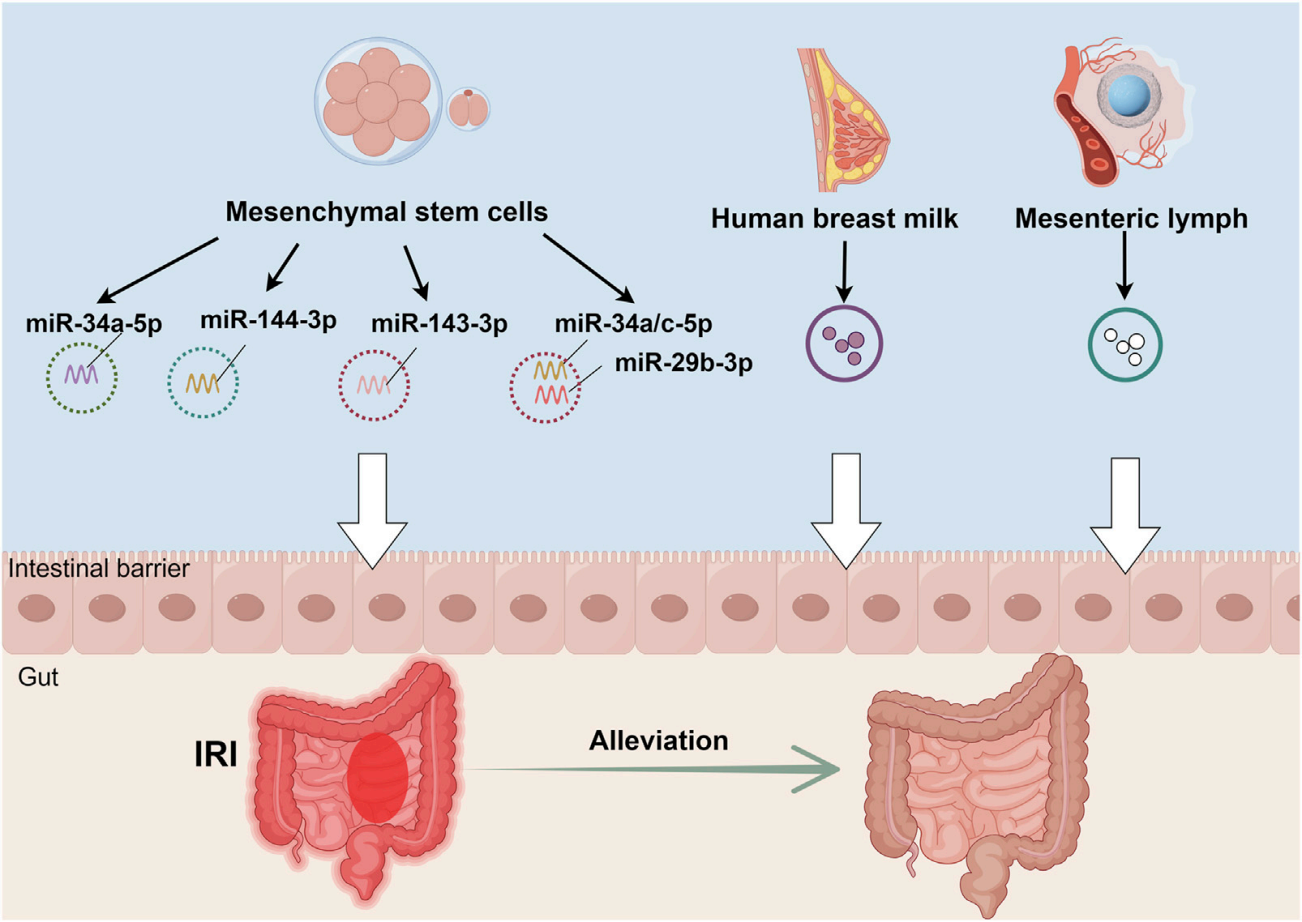
Therapeutic mechanisms of non-intestinal exosomes in intestinal IRI. Exosomes derived from multiple sources - human umbilical cord mesenchymal stem cells, human breast milk, and mesenteric lymph nodes - exhibit protective effects against intestinal IRI through distinct molecular mechanisms. These mechanisms include: (1) activation of the PTEN/Akt/Nrf2 signaling axis, (2) modulation of the TLR4/MyD88/NF-κB inflammatory cascade, and (3) METTL3-dependent pathways. Together, these mechanisms contribute to reduced intestinal cell damage and enhanced barrier function restoration, ultimately attenuating intestinal IRI.

Paralleling intestinal IRI’s systemic effects through exosomes, extra-intestinal exosomes exhibit significant regulatory effects on intestinal IRI, particularly those derived from MSCs. Current evidence indicates that MSC-derived exosomes improve intestinal barrier function by reducing lesion severity, attenuating cellular apoptosis and pyroptosis, and alleviating oxidative stress. These beneficial effects are mediated through modulation of intestinal barrier protein expression and regulation of multiple signaling pathways, including PTEN/Akt/Nrf2, TLR4/MyD88/NF-κB, and METTL3 (Li et al., 2022; Wan et al., 2023; Zhang et al., 2022; Li et al., 2020). Notably, breast milk-derived exosomes demonstrate significant therapeutic potential in intestinal IRI recovery through anti-inflammatory mechanisms and enhanced epithelial cell proliferation (Wang et al., 2022). Moreover, MSC-derived exosomes show promise in treating intestinal IRI-induced remote organ injury, particularly acute lung injury, through inhibition of the TLR4/NF-κB signaling pathway (Liu et al., 2019). Recent investigations have also revealed that mesenteric lymph node exosomes, enriched in polyunsaturated fatty acid-containing lysophosphatidylcholine, activate NF-κB signaling and potentially contribute to intestinal IRI-associated inflammation (Senda et al., 2020). (Table 2; Figure 3)

**TABLE 2:** Effects of non-intestinal exosomes on intestinal IRI.

Exosome origin	Source model	Principal mechanisms and outcomes	References
Mesenchymal stem cells	Mouse	Enhancement of intestinal barrier function via exosomal miR-34a-5p delivery	Li et al. (2022)
Mesenchymal stem cells	Mouse	Attenuation of intestinal injury through miR-143-3p/MyD88-mediated pyroptosis regulation	Wan et al. (2023)
Mesenchymal stem cells	Mouse	Amelioration of oxidative stress-induced intestinal injury via miR-144-3p regulation	Zhang et al. (2022)
Mesenchymal stem cells	Rat	Intestinal barrier protection through exosomal miR-34a/c-5p and miR-29b-3p signaling	Wang et al. (2022)
Mesenchymal stem cells	Rat	Reduction of IRI-induced pulmonary injury via TLR4/NF-κB pathway modulation	Liu et al. (2019)
Human breast milk	Neonatal rat	Protective effects against neonatal intestinal IRI	Wang et al. (2022)
Mesenteric lymph	Rat	Exosomal lipid mediator-induced inflammatory response following intestinal IRI	Senda et al. (2020)

Abbreviations: MyD88, myeloid differentiation factor 88; NF-κB, nuclear factor kappa-light-chain-enhancer of activated B cells; TLR4, toll-like receptor 4.

These findings elucidate the unique regulatory capabilities of extra-intestinal exosomes in intestinal IRI pathophysiology. MSC-derived exosomes, in particular, demonstrate significant protective effects both locally and systemically, highlighting their potential in multi-organ protection. Future research directions should focus on elucidating the temporal dynamics of exosome-mediated effects, particularly in the context of the gut-brain axis, and investigating whether central nervous system-derived exosomes can modulate intestinal IRI progression. Such investigations will enhance our understanding of extra-intestinal exosome function in intestinal IRI and may lead to novel therapeutic strategies for improving patient outcomes.

## 7 Conclusions and perspectives

Intestinal IRI represents a persistent and significant challenge in surgical medicine. Bibliometric analysis reveals substantial research progress in this field from 2004 to 2022, with 1,069 published research articles and reviews demonstrating consistent growth in both volume and citation impact. This collective scholarly effort, contributed by 985 research institutions across 46 countries, underscores the global significance of intestinal IRI research. China and the United States have emerged as leading contributors, demonstrating exceptional achievements in both quantitative output and qualitative depth. Despite these advances, critical challenges persist, necessitating further investigation into cell death mechanisms, exosome functionality, gut microbiota dynamics, and anesthetic effects on intestinal IRI (Wan et al., 2022).

Exosomes, characterized as specialized extracellular vesicles with an approximate diameter of 100 nm, have emerged as crucial mediators in intestinal IRI pathophysiology. These nanovesicles contain diverse molecular cargo, including nucleic acids, proteins, lipids, amino acids, and metabolic byproducts, serving essential functions in cellular homeostasis, intercellular communication, and potential therapeutic applications (Cocucci and Meldolesi, 2015; Bebelman et al., 2018; Willms et al., 2018; Ding et al., 2024; Pathan et al., 2019; van Balkom et al., 2015). Beyond their role in cellular waste management, exosomes function as sophisticated intercellular communication vehicles and promising diagnostic and therapeutic vectors (Kalluri and LeBleu, 2020). Recent evidence has established significant correlations between exosomal activity and IRI pathophysiology across multiple organ systems, particularly in cerebral, cardiac, renal, hepatic, and pulmonary tissues (Liu et al., 2024; Gao et al., 2024; You et al., 2024; Xin et al., 2022).

Intestinal-derived exosomes exhibit complex dual functionality in the pathophysiology of intestinal IRI and subsequent remote organ dysfunction. Upon intestinal IRI onset, these exosomes translocate via the circulatory system to hepatic tissues, where they orchestrate M1 macrophage polarization—a critical process in inflammatory response modulation (Zhao et al., 2022). Moreover, these exosomes demonstrate the capacity to initiate microglial activation, precipitating a cascade of neurological sequelae, including widespread neuronal degeneration, compromised synaptic integrity, and cognitive impairment, thus significantly disrupting neurological homeostasis (Chen et al., 2021). Following intestinal IRI, intestinal epithelial cells augment neurological injury through the secretion of paracrine mediators, particularly exosomal miRNAs that regulate multiple cell death pathways—including apoptosis, necroptosis, and pyroptosis—ultimately culminating in cortical neuronal death (Hsu et al., 2020). Paradoxically, despite these deleterious effects, post-IRI intestinal-derived exosomes exhibit significant therapeutic potential in mitigating intestinal IRI itself. Specifically, intestinal epithelial cell-derived exosomes effectively attenuate hypoxia-high glucose-induced cellular injury and substantially reduce post-IRI intestinal damage severity (Yang et al., 2022). Recent molecular studies have elucidated the pivotal role of exosomal circEZH2_005 in intestinal IRI pathophysiology. This molecular entity functions both as a sensitive diagnostic and prognostic biomarker and as a therapeutic mediator through Gprc5a-dependent mechanisms, suggesting novel therapeutic approaches (Zhang et al., 2023). Furthermore, extra-intestinal exosomes, particularly those derived from MSCs, demonstrate remarkable efficacy in modulating intestinal IRI and potentially influence functional outcomes in affected remote organs, representing a promising therapeutic avenue (Li et al., 2022; Wan et al., 2023; Zhang et al., 2022; Li et al., 2020).

These findings establish exosomes as pivotal mediators in the complex pathophysiological interplay between intestinal IRI and secondary organ dysfunction. Their biological effects are messenger-specific, manifesting in diverse pathophysiological outcomes across affected organ systems. Emerging evidence indicates that exosomal miRNAs facilitate crucial inter-organ communication during IRI events, spanning cerebral, myocardial, renal, and hepatic tissues, thereby providing novel insights into IRI-associated inter-organ interactions (Xin et al., 2022). The therapeutic applications of exosomes extend beyond conventional approaches, encompassing both diagnostic biomarkers and sophisticated drug delivery systems. Integration of intestinal or MSC-derived exosomes in targeted therapeutic strategies may potentiate treatment efficacy through synergistic mechanisms (Zhang et al., 2020). Contemporary investigations further demonstrate the expanded therapeutic potential of exosomes, extending beyond targeted IRI intervention to applications in ischemic preconditioning protocols. These findings present innovative strategies for attenuating perioperative organ injury, with significant implications for clinical practice (Owen et al., 2023).

Despite the promising therapeutic potential of exosomes in intestinal IRI, significant challenges remain in translating these findings into clinical applications. A primary obstacle involves achieving precise targeting of exosomes to specific tissues and cell types. While natural tropism for certain tissues exists, developing strategies for enhanced tissue-specific delivery remains critical for therapeutic efficacy (Wiklander et al., 2015). Current approaches exploring surface modification with targeting ligands and antibodies demonstrate promise but require further optimization to minimize off-target effects and improve therapeutic precision (Murphy et al., 2019).

Bioavailability presents another significant challenge, as exosomes must maintain structural integrity and functional activity following administration. Factors including rapid clearance by the mononuclear phagocyte system, limited stability in circulation, and potential degradation by proteolytic enzymes significantly impact therapeutic efficacy (Antimisiaris et al., 2018). Additionally, the blood-brain barrier presents a formidable obstacle for exosome-based neurotherapeutics in addressing intestinal IRI-induced cognitive impairment, necessitating specialized delivery strategies.

Standardization and scalability represent critical considerations for clinical translation. The heterogeneity of exosome populations, even from identical cellular sources, complicates reproducibility and regulatory compliance. Current isolation and characterization methodologies demonstrate significant variability, potentially affecting therapeutic outcomes (Théry et al., 2018). Furthermore, large-scale production of clinical-grade exosomes with consistent quality and potency remains technically challenging and cost-intensive. Establishing standardized protocols for exosome isolation, characterization, and functional assessment is essential for regulatory approval and successful clinical implementation (Lener et al., 2015).

Finally, the complex cargo composition of exosomes presents both opportunities and challenges. While this molecular diversity contributes to their therapeutic efficacy, it also introduces potential safety concerns, including immunogenicity and off-target effects. Comprehensive understanding of exosomal cargo composition and its functional implications remains essential for optimizing therapeutic applications and ensuring patient safety.

The intestine, recognized as the “second genome” due to its unique microbiome and metabolome, occupies a central position in multi-organ injury pathophysiology (Zhu et al., 2010). This recognition has catalyzed increased attention from both academic researchers and clinicians. Future investigations should prioritize elucidating the mechanistic duality of exosomes in intestinal IRI pathophysiology. Enhanced understanding of these complex molecular interactions will not only advance our knowledge of exosome biology in intestinal IRI but also facilitate the development of innovative therapeutic strategies to improve clinical outcomes in affected patients.

## Abbreviations

ESE, endosome; EVs, extracellular vesicles; ILVs, intraluminal vesicles; IRI, ischemia-reperfusion injury; LSEs, late sorting endosomes; MSC, mesenchymal stem cell; MVBs, multivesicular bodies.

## References

[B1] Antimisiaris S. G. Mourtas S. Marazioti A. (2018). Exosomes and exosome-inspired vesicles for targeted drug delivery. Pharmaceutics 10 (4), 218. 10.3390/pharmaceutics10040218 30404188 PMC6321407

[B2] Bebelman M. P. Smit M. J. Pegtel D. M. Baglio S. R. (2018). Biogenesis and function of extracellular vesicles in cancer. Pharmacol. Ther. 188, 1–11. 10.1016/j.pharmthera.2018.02.013 29476772

[B3] Cai X. Zhang Z. Y. Yuan J. T. Ocansey D. K. W. Tu Q. Zhang X. (2021). hucMSC-derived exosomes attenuate colitis by regulating macrophage pyroptosis via the miR-378a-5p/NLRP3 axis. Stem Cell Res. Ther. 12 (1), 416. 10.1186/s13287-021-02492-6 34294138 PMC8296541

[B4] Chen X. D. Zhao J. Yang X. Zhou B. W. Yan Z. Liu W. F. (2021). Gut-derived exosomes mediate memory impairment after intestinal ischemia/reperfusion via activating Microglia. Mol. Neurobiol. 58 (10), 4828–4841. 10.1007/s12035-021-02444-4 34189701

[B5] Cocucci E. Meldolesi J. (2015). Ectosomes and exosomes: shedding the confusion between extracellular vesicles. Trends Cell Biol. 25 (6), 364–372. 10.1016/j.tcb.2015.01.004 25683921

[B6] Ding S. Kim Y. J. Huang K. Y. Um D. Jung Y. Kong H. (2024). Delivery-mediated exosomal therapeutics in ischemia-reperfusion injury: advances, mechanisms, and future directions. Nano Converg. 11 (1), 18. 10.1186/s40580-024-00423-8 38689075 PMC11061094

[B7] Ding Z. Zhang C. Zhang B. Li Q. (2022). Unraveling the proteomic Landscape of intestinal epithelial cell-derived exosomes in mice. Front. Physiol. 13, 773671. 10.3389/fphys.2022.773671 35283765 PMC8905357

[B8] Gao C. Chen L. Xie X. Y. He X. F. Shen J. Zheng L. (2024). Bone marrow mesenchymal stem cells-derived exosomal miR-381 alleviates lung ischemia-reperfusion injury by activating Treg differentiation through inhibiting YTHDF1 expression. Cell Signal 124, 111440. 10.1016/j.cellsig.2024.111440 39357613

[B9] Gao C. Zhou Y. Chen Z. Li H. Xiao Y. Hao W. (2022). Turmeric-derived nanovesicles as novel nanobiologics for targeted therapy of ulcerative colitis. Theranostics 12 (12), 5596–5614. 10.7150/thno.73650 35910802 PMC9330521

[B10] Hsu C. C. Huang C. C. Chien L. H. Lin M. T. Chang C. P. Lin H. J. (2020). Ischemia/reperfusion injured intestinal epithelial cells cause cortical neuron death by releasing exosomal microRNAs associated with apoptosis, necroptosis, and pyroptosis. Sci. Rep. 10 (1), 14409. 10.1038/s41598-020-71310-5 32873851 PMC7462997

[B11] Jiménez-Castro M. B. Cornide-Petronio M. E. Gracia-Sancho J. Peralta C. (2019). Inflammasome-mediated inflammation in liver ischemia-reperfusion injury. Cells 8 (10), 1131. 10.3390/cells8101131 31547621 PMC6829519

[B12] Kalluri R. LeBleu V. S. (2020). The biology, function, and biomedical applications of exosomes. Science 367 (6478), eaau6977. 10.1126/science.aau6977 32029601 PMC7717626

[B13] Kim J. Zhang S. Zhu Y. Wang R. Wang J. (2023). Amelioration of colitis progression by ginseng-derived exosome-like nanoparticles through suppression of inflammatory cytokines. J. Ginseng Res. 47 (5), 627–637. 10.1016/j.jgr.2023.01.004 37720571 PMC10499592

[B14] Kojima M. Costantini T. W. Eliceiri B. P. Chan T. W. Baird A. Coimbra R. (2018). Gut epithelial cell-derived exosomes trigger posttrauma immune dysfunction. J. Trauma Acute Care Surg. 84 (2), 257–264. 10.1097/TA.0000000000001748 29194317

[B15] Lener T. Gimona M. Aigner L. Börger V. Buzas E. Camussi G. (2015). Applying extracellular vesicles based therapeutics in clinical trials - an ISEV position paper. J. Extracell. Vesicles 4, 30087. 10.3402/jev.v4.30087 26725829 PMC4698466

[B16] Lewis C. V. Taylor W. R. (2020). Intestinal barrier dysfunction as a therapeutic target for cardiovascular disease. Am. J. Physiol. Heart Circ. Physiol. 319 (6), H1227–H1233. 10.1152/ajpheart.00612.2020 32986965 PMC7792706

[B17] Li Y. J. Xu Q. W. Xu C. H. Li W. M. (2022). MSC promotes the secretion of exosomal miR-34a-5p and improve intestinal barrier function through METTL3-mediated pre-miR-34a m6A modification. Mol. Neurobiol. 59 (8), 5222–5235. 10.1007/s12035-022-02833-3 35687301

[B18] Li Y. Y. Xu Q. W. Xu P. Y. Li W. M. (2020). MSC-derived exosomal miR-34a/c-5p and miR-29b-3p improve intestinal barrier function by targeting the Snail/Claudins signaling pathway. Life Sci. 257, 118017. 10.1016/j.lfs.2020.118017 32603821

[B19] Liao S. Luo J. Kadier T. Ding K. Chen R. Meng Q. (2022). Mitochondrial DNA release contributes to intestinal ischemia/reperfusion injury. Front. Pharmacol. 13, 854994. 10.3389/fphar.2022.854994 35370747 PMC8966724

[B20] Lier H. Bernhard M. Hossfeld B. (2018). [Hypovolemic and hemorrhagic shock]. Anaesthesist 67 (3), 225–244. 10.1007/s00101-018-0411-z 29404656

[B21] Liu J. Chen T. Lei P. Tang X. Huang P. (2019). Exosomes released by bone marrow mesenchymal stem cells attenuate lung injury induced by intestinal ischemia reperfusion via the TLR4/NF-κB pathway. Int. J. Med. Sci. 16 (9), 1238–1244. 10.7150/ijms.35369 31588189 PMC6775266

[B22] Liu Y. Chen J. Xiong J. Hu J. Q. Yang L. Y. Sun Y. X. (2024). Potential cardiac-derived exosomal miRNAs involved in cardiac healing and remodeling after myocardial ischemia-reperfusion injury. Sci. Rep. 14 (1), 24275. 10.1038/s41598-024-75517-8 39414956 PMC11484883

[B23] Miyake H. Lee C. Chusilp S. Bhalla M. Li B. Pitino M. (2020). Human breast milk exosomes attenuate intestinal damage. Pediatr. Surg. Int. 36 (2), 155–163. 10.1007/s00383-019-04599-7 31713717

[B24] Murphy D. E. de Jong O. G. Brouwer M. Wood M. J. Lavieu G. Schiffelers R. M. (2019). Extracellular vesicle-based therapeutics: natural versus engineered targeting and trafficking. Exp. Mol. Med. 51 (3), 1–12. 10.1038/s12276-019-0223-5 PMC6418170 30872574

[B25] Owen A. Stary C. M. Gross E. R. (2023). Exosomes as perioperative therapeutics to limit organ injury. Br. J. Anaesth. 130 (3), 248–250. 10.1016/j.bja.2022.12.014 36682935

[B26] Pathan M. Fonseka P. Chitti S. V. Kang T. Sanwlani R. Van Deun J. (2019). Vesiclepedia 2019: a compendium of RNA, proteins, lipids and metabolites in extracellular vesicles. Nucleic Acids Res. 47 (D1), D516–D519. 10.1093/nar/gky1029 30395310 PMC6323905

[B27] Senda A. Kojima M. Watanabe A. Kobayashi T. Morishita K. Aiboshi J. (2023). Profiles of lipid, protein and microRNA expression in exosomes derived from intestinal epithelial cells after ischemia-reperfusion injury in a cellular hypoxia model. PLoS One 18 (3), e0283702. 10.1371/journal.pone.0283702 36989330 PMC10058167

[B28] Senda A. Morishita K. Kojima M. Doki S. Taylor B. Yagi M. (2020). The role of mesenteric lymph exosomal lipid mediators following intestinal ischemia-reperfusion injury on activation of inflammation. J. Trauma Acute Care Surg. 89 (6), 1099–1106. 10.1097/TA.0000000000002897 32769950

[B29] Sunder-Plassmann P. (1952). [Reperfusion injury and its treatment]. Dermatol Wochenschr 125 (10), 224–234. 14945272

[B30] Teng Y. Ren Y. Sayed M. Hu X. Lei C. Kumar A. (2018). Plant-derived exosomal MicroRNAs Shape the gut microbiota. Cell Host Microbe 24 (5), 637–652. 10.1016/j.chom.2018.10.001 30449315 PMC6746408

[B31] Théry C. Witwer K. W. Aikawa E. Alcaraz M. J. Anderson J. D. Andriantsitohaina R. (2018). Minimal information for studies of extracellular vesicles 2018 (MISEV2018): a position statement of the International Society for Extracellular Vesicles and update of the MISEV2014 guidelines. J. Extracell. Vesicles 7 (1), 1535750. 10.1080/20013078.2018.1535750 30637094 PMC6322352

[B32] van Balkom B. W. Eisele A. S. Pegtel D. M. Bervoets S. Verhaar M. C. (2015). Quantitative and qualitative analysis of small RNAs in human endothelial cells and exosomes provides insights into localized RNA processing, degradation and sorting. J. Extracell. Vesicles 4, 26760. 10.3402/jev.v4.26760 26027894 PMC4450249

[B33] Wan Y. Dong P. Zhu X. Lei Y. Shen J. Liu W. (2022). Bibliometric and visual analysis of intestinal ischemia reperfusion from 2004 to 2022. Front. Med. (Lausanne) 9, 963104. 10.3389/fmed.2022.963104 36052333 PMC9426633

[B34] Wan Z. Zhang Y. Lv J. Yuan Y. Guo W. Leng Y. (2023). Exosomes derived from bone marrow mesenchymal stem cells regulate pyroptosis via the miR-143-3p/myeloid differentiation factor 88 axis to ameliorate intestinal ischemia-reperfusion injury. Bioengineered 14 (1), 2253414. 10.1080/21655979.2023.2253414 37674357 PMC10486297

[B35] Wang L. Gao R. Li B. Alganabi M. He W. Shen C. (2022). Human breast milk-derived exosomes protect against intestinal ischemia and reperfusion injury in neonatal rats. J. Pediatr. Surg. 57 (7), 1264–1268. 10.1016/j.jpedsurg.2022.02.029 35379491

[B36] Wei S. Wu X. Chen M. Xiang Z. Li X. Zhang J. (2023). Exosomal-miR-129-2-3p derived from Fusobacterium nucleatum-infected intestinal epithelial cells promotes experimental colitis through regulating TIMELESS-mediated cellular senescence pathway. Gut Microbes 15 (1), 2240035. 10.1080/19490976.2023.2240035 37550944 PMC10411316

[B37] Wiklander O. P. Nordin J. Z. O'Loughlin A. Gustafsson Y. Corso G. Mäger I. (2015). Extracellular vesicle *in vivo* biodistribution is determined by cell source, route of administration and targeting. J. Extracell. Vesicles 4, 26316. 10.3402/jev.v4.26316 25899407 PMC4405624

[B38] Willms E. Cabañas C. Mäger I. Wood M. J. A. Vader P. (2018). Extracellular vesicle heterogeneity: Subpopulations, isolation Techniques, and diverse functions in cancer progression. Front. Immunol. 9, 738. 10.3389/fimmu.2018.00738 29760691 PMC5936763

[B39] Wu M. Y. Yiang G. T. Liao W. T. Tsai A. P. Cheng Y. L. Cheng P. W. (2018). Current mechanistic Concepts in ischemia and reperfusion injury. Cell Physiol. Biochem. 46 (4), 1650–1667. 10.1159/000489241 29694958

[B40] Xin W. Qin Y. Lei P. Zhang J. Yang X. Wang Z. (2022). From cerebral ischemia towards myocardial, renal, and hepatic ischemia: exosomal miRNAs as a general concept of intercellular communication in ischemia-reperfusion injury. Mol. Ther. Nucleic Acids 29, 900–922. 10.1016/j.omtn.2022.08.032 36159596 PMC9464648

[B41] Yang J. Zheng X. G. Wu Y. L. Wang A. P. Wang C. H. Chen W. X. (2022). Intestinal epithelial cell-derived exosomes package microRNA-23a-3p alleviate gut damage after ischemia/reperfusion via targeting MAP4K4. Biomed. Pharmacother. 149, 112810. 10.1016/j.biopha.2022.112810 35303564

[B42] You Y. Chen S. Tang B. Xing X. Deng H. Wu Y. (2024). Exosome-related gene identification and diagnostic model construction in hepatic ischemia-reperfusion injury. Sci. Rep. 14 (1), 22450. 10.1038/s41598-024-73441-5 39341981 PMC11439056

[B43] Zhang G. Wan Z. Liu Z. Liu D. Zhao Z. Leng Y. (2022). Exosomes derived from BMSCs ameliorate intestinal ischemia-reperfusion injury by regulating miR-144-3p-mediated oxidative stress. Dig. Dis. Sci. 67 (11), 5090–5106. 10.1007/s10620-022-07546-0 35624329

[B44] Zhang H. Wang L. Li C. Yu Y. Yi Y. Wang J. (2019). Exosome-induced regulation in inflammatory bowel disease. Front. Immunol. 10, 1464. 10.3389/fimmu.2019.01464 31316512 PMC6611439

[B45] Zhang W. Zhou B. Yang X. Zhao J. Hu J. Ding Y. (2023). Exosomal circEZH2_005, an intestinal injury biomarker, alleviates intestinal ischemia/reperfusion injury by mediating Gprc5a signaling. Nat. Commun. 14 (1), 5437. 10.1038/s41467-023-41147-3 37673874 PMC10482849

[B46] Zhang Y. Bi J. Huang J. Tang Y. Du S. Li P. (2020). Exosome: a review of its classification, isolation Techniques, storage, diagnostic and targeted therapy applications. Int. J. Nanomedicine 15, 6917–6934. 10.2147/IJN.S264498 33061359 PMC7519827

[B47] Zhao J. Chen X. D. Yan Z. Z. Huang W. F. Liu K. X. Li C. (2022). Gut-derived exosomes induce liver injury after intestinal ischemia/reperfusion by promoting hepatic macrophage polarization. Inflammation 45 (6), 2325–2338. 10.1007/s10753-022-01695-0 35701685

[B48] Zhu B. Wang X. Li L. (2010). Human gut microbiome: the second genome of human body. Protein Cell 1 (8), 718–725. 10.1007/s13238-010-0093-z 21203913 PMC4875195

